# Supplementary material for: Probing the Cellular Fate of the Protein Corona around Nanoparticles with Nanofocused X-ray Fluorescence Imaging
Source: Int J Mol Sci. 2023 Dec 30;25(1):528. doi: 10.3390/ijms25010528 (PMC10778884; doi:10.3390/ijms25010528)
Supplement: Supplementary file 1 [file ijms-25-00528-s001.zip › ijms-2777389-supplementary.pdf]

International Journal of *Molecular Sciences*

MDPI

Supporting Information

Probing the cellular fate of the protein corona around nanoparticles with nanofocused X-ray fluorescence imaging

Marvin Skiba[1,2], Gabriela Guedes[3], Dmitry Karpov[4], Neus Feliu[5], Aitziber L. Cortajarena[3,6], Wolfgang J. Parak[1,2*], Carlos Sanchez-Cano[6,7,8*]

[1] Center for Hybrid Nanostructures, University of Hamburg, 22761 Hamburg, Germany
[2] The Hamburg Centre for Ultrafast Imaging, 22761 Hamburg, Germany
[3] Center for Cooperative Research in Biomaterials (CIC biomaGUNE), Basque Research and Technology Alliance, 20014 Donostia-San Sebastian, Spain
[4] European Synchrotron Radiation Facility, 38000 Grenoble, France
[5] Zentrum für Angewandte Nanotechnologie CAN, Fraunhofer-Institut für Angewandte Polymerforschung IAP, 20146 Hamburg, Germany
[6] Ikerbasque, Basque Foundation for Science, 48013 Bilbao, Spain
[7] Donostia International Physics Center, 20018 Donostia-San Sebastian, Spain
[8] Polimero eta Material Aurreratuak: Fisika, Kimika eta Teknologia, Kimika Fakultatea, Euskal Herriko Unibertsitatea UPV/EHU, 20018 Donostia-San Sebastian, Spain

* Correspondence: CSC: carlos.sanchez@dipc.org; WJP: wolfgang.parak@uni-hamburg.de

S1. Extended methods

S1.1. Gold nanoparticle synthesis

First, a citrate buffer was prepared by mixing 215 mL aqueous sodium citrate (2.75 mM) solution with 71 mL aqueous citric acid (2.75 mM). The solution was boiled for 15 minutes under vigorous stirring, followed by the addition of 2.5 mg ethylenediaminetetraacetic acid (EDTA). Next, 71 mL of hot gold precursor solution ($HAuCl_4$ in water, 812.5 µM) was rapidly added. The reaction mixture was boiled and agitated for 20 minutes and cooled down to room temperature afterwards [29]. The synthesis yielded in ~300 mL NP solution with a concentration of c_{NP} = 1.3 nM, determined from UV-Vis absorbance spectroscopy (see section S2.1).

S1.2. Surface modification of AuNP with HS-PEG-NH_2

<u>Deprotection of Trt-S-PEG-NH_2:</u> 0.0349 mmol of commercially available tritylthiol-PEG-amine (3 kDa) was dissolved in 3.7 mL CH_2Cl, followed by the addition of 307 µL triisopropyl silane (TIPS) and 223 µL triflouroacetic acid (TFA). Consequently, similar amounts of TIPS and TFA were added again. The solution was covered with parafilm and vigorously stirred for 15 minutes. The solvents were evaporated by rotary evaporator (60 °C, 30 mbar) leading to a white solid in the reaction flask. The compound was triturated and filter-washed with diethyl ether. Finally, 0.0296 mmol (yield: 84.8%) were obtained. [53]

Pegylation of AuNP: The so obtained thiol-PEG-amine was dissolved in miliQ water (10 mg/mL) and immediately added to the gold nanoparticle solution (250 PEG/nm^2 gold surface). The total gold surface in solution was calculated by assuming a perfect sphere for each gold nanoparticle, with a diameter of d_c = 12 nm [54]. The reaction was covered by parafilm and stirred overnight at room temperature before collecting in a 40 mL Nalgene centrifuge tubes. First centrifugation was conducted at 4 °C, 30,000 rcf, 60 min. The pellets were resuspended in miliQ water, transferred in 2 mL Eppendorf centrifuge tubes for five more rounds of centrifugation (25,000 rcf, 30 min), while replacing the supernatant with fresh miliQ water to remove unreacted PEG molecules and precursors.

S1.3. Cell culture

Mouse embryonic fibroblasts (3T3) cells were cultured in Alpha modified eagles' medium (AMEM), supplemented with 100 U/mL penicillin-streptomycin and 10 % fetal bovine serum. Cells were grown until a confluency of 70-80% was reached at 37 °C and 5% CO_2. Subculturing was conducted every 2-3 days with 0.05 % Trypsin/EDTA for chemical detachment of adherent cells.

S1.4. Cell viability assay

Cell viability after exposure to AuNPs, CTPR-Gd protein, or the hybrid AuNP@CTPR-Gd was determined through individual MTT assays [55]. For this, 2 × 10^3 cells in 100 µL of AMEM cell media were seeded per well in a 96-well plate and grown overnight. The following day the growth media was removed, followed by exposure to 100 µL fresh growth media, containing the compound of interest in different concentrations. In this study the range was between 0.1 – 50 µg/mL Au, 3 nM – 1.5 µM of free protein, respectively. After 24 h at 37 °C, 5 % CO_2, the cells were washed with 100 µL phosphate buffered saline (PBS) and subsequently 100 µL growth medium including 0.5 mg/mL 3-(4,5-dimethylthiazol-2-yl)-5-(3-carboxymethoxyphenyl)-2-(4-sulfophenyl)-2H-tetrazolium (MTT) was added. Finally, after 2 h incubation, the growth medium was removed, and 100 µL dimethyl sulfoxide (DMSO) was added to dissolve the in situ formed formazan crystals. The absorbance of individual wells was subsequently measured at a fixed wavelength of 550 nm and background-autocorrected by the absorbance at 630 nm.

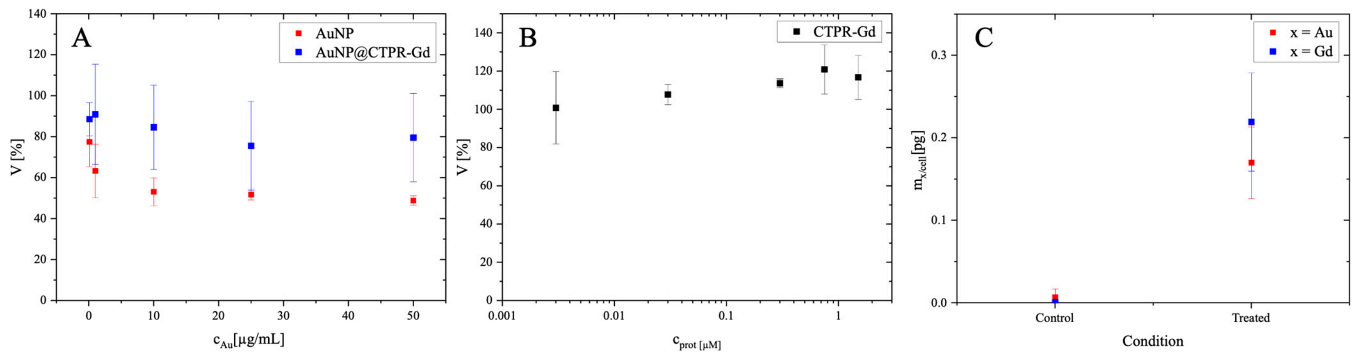

Figure S1 (A) Cell viability V of AuNP and AuNP@CTPR-Gd, and (B) free CTPR-Gd, in dependence of the Au concentration C_{Au} and the protein concentration c_{prot}, respectively. (C) Mass of endocytosed Au and Gd *per* cell ($m_{Au/cell}$, $m_{Gd/cell}$ [pg]) on 3T3 cells exposed to C_{Au} = 0 (control) or C_{Au} = 10 µg/mL AuNP@CTPR-Gd (treated) for 24 h, without following incubation in NP-free medium (t_{inc} = 0) as measured with ICP-MS.

S1.5. Cellular uptake study

Confirmation and quantification of cellular uptake of NPs were carried out by inductively coupled plasma - mass spectrometry (ICP-MS). For this, 3×10^5 cells in 2 mL AMEM growth medium were seeded in 6- well plates and grown for 24 h. After removing the medium, the cells were exposed to AuNP@CTPR-Gd (C_{Au} = 10 µg/mL), carried in 2 mL growth medium. After 24 h exposure the medium was removed, cells were washed with 1 mL PBS before chemical detachment with 500 µL 0.05 % Trypsin/EDTA. Cells were collected by adding 1.5 mL culture medium and transferred in 15 mL falcon tube. Next, trypan blue was used to manually quantify life cells in a Neubauer hemocytometer [56]. Afterwards, the cells were centrifuged (200 rcf, 5 min) and an additional 1 mL of aqua regia was added to digest them using a Berghof Speedwave XPERT microwave digestor. Finally, the amount of Au and Gd was determined by ICP-MS (Thermo Fisher iCAP-Q).

S2. Characterization of hybrid nanoparticles

S2.1. Pegylated gold nanoparticles

The concentration of AuNP was determined using Beer-Lambert law, calculating the molar extinction coefficient based on the NP diameter as obtained by TEM [57]. For the NPs used here with core diameter d_c = 11.9 nm accordingly a molar extinction coefficient (at the plasmon peak) of ε_{NP} = 1.84 $\cdot 10^8$ $M^{-1}cm^{-1}$ was determined. Each AuNP contains around $N_{Au/NP}$ = 51,953 Au atoms [54].

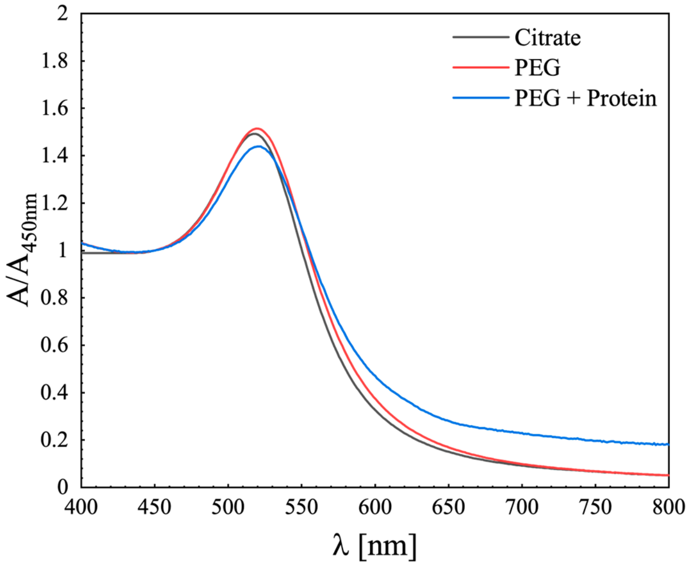

Figure S2 UV-vis absorbance spectra of AuNP with different surface coatings.

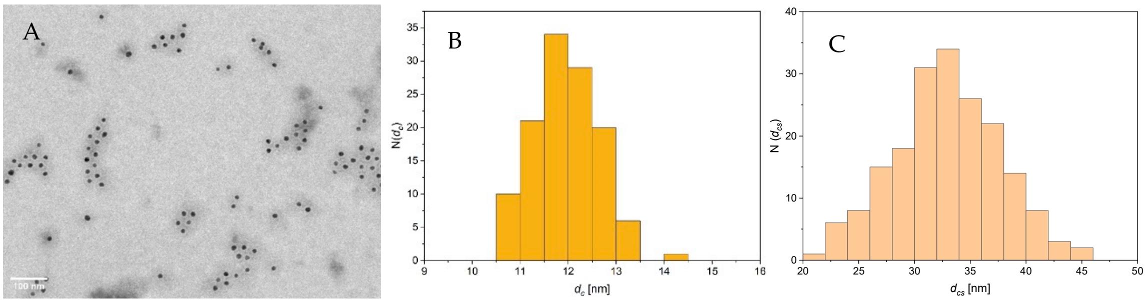

Figure S3 (A) Representative transmission electron microscopy (TEM) image of AuNP with negative staining (with Uranyl acetate), used for PEG coated experiments. The scale bar indicates 100 nm. (B) Histogram of 121 measured NP core sizes (d_c) and (C) histogram of 189 measured NP diameter including organic shell (d_{cs}), based on TEM images.

S2.2. Characterization of the protein corona around pegylated AuNP

The change in hydrodynamic diameter d_h, measured by dynamic light scattering (DLS), after incubating the AuNP with 225 equivalents of CTPR-Gd protein for 2 h at 37 °C didn't show an increment in size (d_h). However, a wavelength shift of 1 nm was observed by UV-Vis absorption spectroscopy (Fig. S1). Additionally, an indirect method using ICP-MS was conducted. For this, the initial ratio between CTPR-Gd protein and AuNP was determined to be 228. After three rounds of centrifugation (20 krcf, 30 min), changing the supernatant with PBS, still a ratio of 128 was detected. Since commonly this centrifugation parameters are not sufficient to pellet proteins, we conclude a "hard" protein corona was formed [58]. Moreover, gel electrophoresis was conducted with pegylated AuNP (left lane) and the mixture of AuNP@CTPR-Gd (right lane). It is shown that the pure AuNP moved towards the anode, driven by the amino group moieties on the PEG. In contrast the AuNP with CTPR-Gd protein (AuNP@CTPR-Gd) stuck in the pocket and even concentrated slightly in direction in the direction of the cathode, which can be explained by the negative surface potential of the protein on top of positively charged pegylated AuNP.

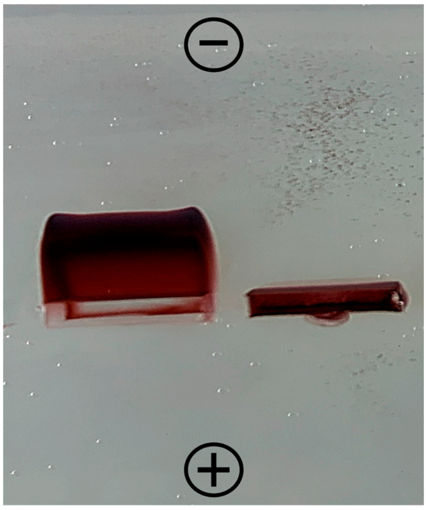

Figure S4 Agarose Gel electrophoresis (2% agarose) of AuNP (left lane) and AuNP@CTPR-Gd (right lane).

S3. Protein characterization

S3.1. Protein design, cloning, and molecular biology

The consensus tetratricopeptide repeat (CTPR) protein used herein was engineered using a cloning approach previously described [59], and is constituted by 11 repeats from which six were engineered to display 4 Cys at non-conserved residues (C4Cys) for metal coordination [60]. The gene encoding this protein was constructed temploying a block cloning strategy that involved BamHI and BglII digestion [9]. This gene was

then cloned into the pProEx-HTA vector coding for N-terminal hexahistidine tag and resistance to ampicillin. Finally, the gene sequence was checked by DNA sequencing (Stab Vida).

S3.2. Protein expression and purification

The vector containing the engineered CTPR gene was transformed into Escherichia coli C41. Afterwards, CTPR protein was expressed by growing the bacteria at 37 °C until the culture reached an optical density in the range 0.6-0.8. The protein expression was then induced with 1 mM of isopropyl β-d-thiogalactoside (IPTG) followed by 24 h growth at 20 ˚C. Subsequently, the bacterial cells were harvested through centrifugation at 4500 rpm and 20 ˚C for 15 min. The resulting cell pellet was reconstituted in a buffer solution containing 500 mM sodium chloride, 500 mM urea, 50 mM Tris at pH 8.0, and then subjected to lysis via a freezing-thaw cycle and sonication. The obtained lysate was subsequently centrifuged at 10000 rpm, 4 ˚C for 1 h. The supernatant was purified using affinity chromatography, specifically the HisTrap™ High Performance, Cytiva. Following this, the hexahistidine tag was cleaved using Tobacco Etch Virus (TEV) protease. The mixture of CTPR protein, TEV, and hexahistidine tag was dialyzed against Tris buffer using a dialysis membrane with molecular weight cutoff of 6-8 KDa (SpectraPor®), to eliminate the imidazole. Subsequently, the solution underwent another round of purification through affinity chromatography to remove the TEV and hexahistidine tag from the protein solution. The concentration of protein was determined by measuring UV absorbance at 280 nm and applying the Beer-Lambert law (ε = 148420 M^{-1} cm^{-1}).

The expression and purification of the engineered CTPR protein was verified through the acrylamide gel and by MALDI-ToF (Figure S5). The mass spectrum was acquired on an Applied Biosystems Voyager Elite MALDI-TOF mass spectrometer with delayed extraction (Applied Biosystems, Framingham, MA, USA) equipped with a pulsed N2 laser (λ= 337 nm). The mass spectra was acquired in positive reflection mode using delayed extraction using an extraction voltage of 20 kV. The sample deposition was prepared as follows: 1 µL of sample was mixed in a stainless steel MALDI target with 4 µL synapinic acid matrix and let dry before analysis. The molecular weight obtained experimentally by MALDI-ToF (45478 Da) is in agreement with the theoretically determined from the protein sequence using ExPASy ProtParam tool (45533 Da).

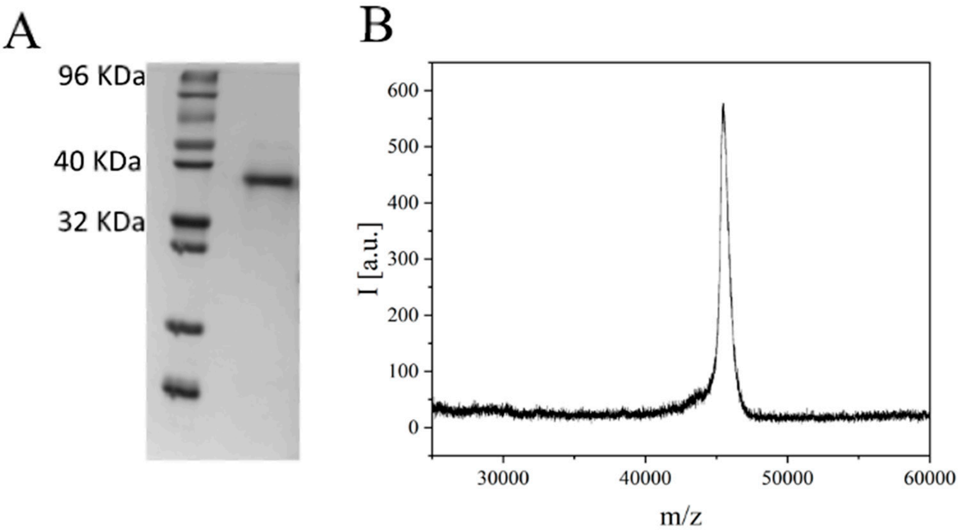

Figure S5 CTPR protein characterization. (A) Acrylamide gel (12% acrylamide) of the purified CTPR protein (protein marker on the left). (B) MALDI-ToF spectrum of the CTPR protein, showing the relative intensity distribution I of the mass to charge ration m/z.

S3.3. Labelling of CTPR protein with Gd

CTPR protein was labelled with Gd following an adapted biomineralization-like procedure [58]. In brief, 1 mL of protein at 20 mM was incubated with $GdCl_3$ solution (in the ratio 1:6 of protein to gadolinium metals) for 30 min at 37 ˚C and 800 rpm. Subsequently, 1.2 mL of NaOH (1 M) were added and further incubated overnight at 37 ˚C and 800 rpm. The CTPR-Gd was then purified using a PD-10 column to separate the labelled protein from unreacted metals and to buffer exchange to PBS. Finally, the CTPR-Gd was stored at 4 ˚C until further use. The protein concentration in the final sample was determined by Bradford assay (Thermofisher). The presence of Gd was confirmed by ICP-MS, obtaineing an average of 6 atoms of Gd per protein.

S4. Colocalization

S4.1. Colocalization analysis

Colocalization describes an overlap of variables in the same place. This is, of course, more complex than it seems at first glance. In microscopy, resolution is limited to a certain pixel size, given by physical or even technical parameters. For example, signals from different channels are fully colocalized when measured in one pixel. On the other hand, the Pauli principle rules out complete colocalization of two objects, since not all quantum numbers can be the same. This means that a meaningful analysis is located between these two

extremes. For this work, we need to further distinguish between co-occurrence - the overlap of two signals with respect to their pixel intensities - and correlation - the relationship between the signal intensities in one pixel and the corresponding values in another. To match and analyze the co-occurrence of signals the Pearsons's correlation coefficient r as well as Mander's coefficients M_1 and M_2 were determined for all XRF maps using the Coloc2 plugin in the Fiji software V. 1.53c [52]. For that, regions of interest were chosen, based on the K K_α emission line, indicating cellular structure.

S4.2. Pearson's correlation coefficient (*PCC, r*)

A golden standard for matching and predicting the relationship between two types of patterns was described mathematically by Pearson in 1896 (Equation 1). The *PCC* can be thought of as the covariance - relationship between two random variables with a common probability distribution - between the two images, which is then normalized to the product of the individual standard deviations [9].

$$r = \frac{\sum_i (I_A(i) - <I_A>) \cdot (I_B(i) - <I_B>)}{\sqrt{[\sum_i (I_A(i) - <I_A>)^2 \cdot \sum_i (I_B(i) - <I_B>)^2]}} \quad \text{(Equ. 1)}$$

$I_A(i)$ and $I_B(i)$ represent the intensities (here grayscale values) of the different channels (A, B) in the pixel *i*, while $<I_A>$ and $<I_B>$ are the mean values, respectively. The *PCC* itself can be a value in the range -1 to 1, where -1 means perfect anti-correlation, 0 means no correlation (random distribution), and 1 means perfect correlation. Note that the calculation is not sensitive to differences in mean signal intensity but is affected in the case of a poor signal-to-noise ratio, pushing the value close to 0 [61,62].

S4.3. Manders' coefficients (M_x)

Another method of determining the overlap of two signals, considering pixel intensities, was introduced by Manders in 1993. Here, the co-occurrence (or abundance) is expressed by two different coefficients: M_1 - the proportion of signal from channel *A* in a pixel (*i*) that overlaps with signal from channel *B* in the same pixel above a certain threshold, M_2 correspondingly *vice versa* [63].

$$M_1 = \frac{\sum_i I_A(i)_{coloc}}{\sum_i I_A(i)} \quad \text{with: } I_A(i)_{coloc} = \begin{Bmatrix} I_A(i): I_B(i)>0 \\ 0: I_B(i)=0 \end{Bmatrix} \quad \text{(Equ. 2)}$$

And:

$$M_2 = \frac{\sum_i I_B(i)_{coloc}}{\sum_i I_B(i)} \quad \text{with: } I_B(i)_{coloc} = \begin{Bmatrix} I_B(i): I_A(i)>0 \\ 0: I_A(i)=0 \end{Bmatrix} \quad \text{(Equ. 3)}$$

The Manders coefficients are thus inherently quite insensitive to low signal-to-noise ratios since many pixels are summed above the specified threshold and thus the influence of random noise distribution is negligible.

The values range from 0 to 1 and often differ from each other, which provides the opportunity to investigate individual dependencies [63].

S4.4. Simulation of test patterns – Costes' significance

To test the analysis procedure, Costes introduced a statistically significant algorithm based on Pearsons' correlation coefficient. Costes' significant test is based on randomly scrambling each pixel in an image and comparing the colocalization to the other, unaltered image. After the PCC is generated for a predetermined number of randomly generated images (about 200), the proportion of PCCs that is lower than the initial value (Costes' P value) is calculated. If this value is ≥ 0.95, it indicates significant true colocalization [65].

For clarification and better understanding of the different coefficients, some examples are shown below (Fig. S6). The images were created manually and converted to grayscale images before analysis.

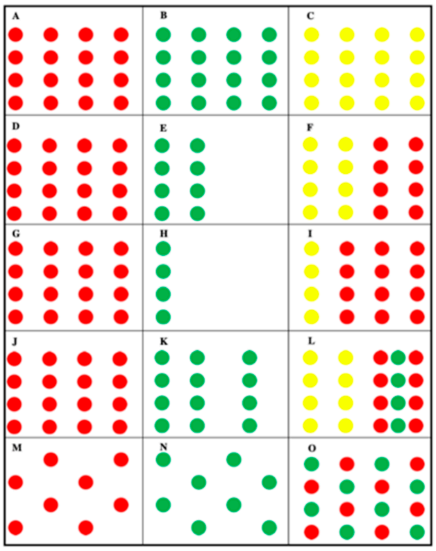

Figure S6 Simulated test patterns for calculating colocalization coefficients. D, G, J are copies from A to better demonstrate merged images *per* row. C, F, I, L, O are the merged images made of the respective first and second row.

Table S1 Persons' correlation coefficient (*r*), Manders's coefficients (M_1, M_2) and Costes P-value generated on the simulated test patterns (Fig. S6).

**Images**	***r***	M_1	M_2	***P***
A + B	1	1	1	1.00
D + E	0.70	0.5	1	1.00
G + H	0.49	0.25	1	1.00
J + K	0.55	0.5	0.67	1.00
M + N	-1	0	0	0.00

This illustrates the important role of Manders coefficients, *e.g.*: when analyzing the colocalization between Figure S6 J and K, the PCC value of r = 0.55 is difficult to interpret, while the situation with the Manders coefficients is clear. The values confirm that half of the "red" signal co-occurs with the "green" signal and two-thirds of the "green" signal co-occurs with the "red" signal, just as exemplified in the merged Figure S6 L.

S5. Elemental XRF-imaging of all investigated cells

S5.1. X-ray fluorescence spectra

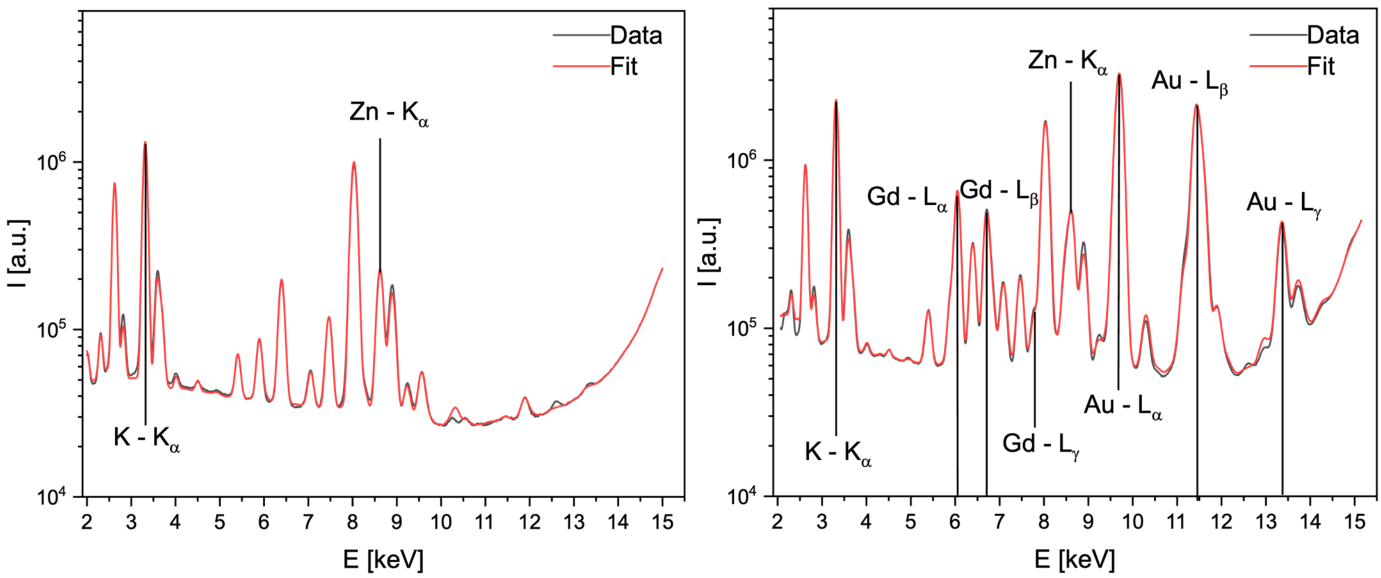

Figure S7 Summed X-ray fluorescence spectra of 3T3 cell mapping, showing the detected intensity I versus energy E. Left spectra (control in which cells were not exposed to AuNP@CTPR-Gd) shows no sign of Au or Gd L_α emissions as expected, whereas in the AuNP@CTPR-Gd treated sample (right) L_α emissions from Gd (*e.g.* 6.7 keV) and Au (*e.g.* 9.7 or 11.6 keV) are clearly visible.

S5.2. Different elements

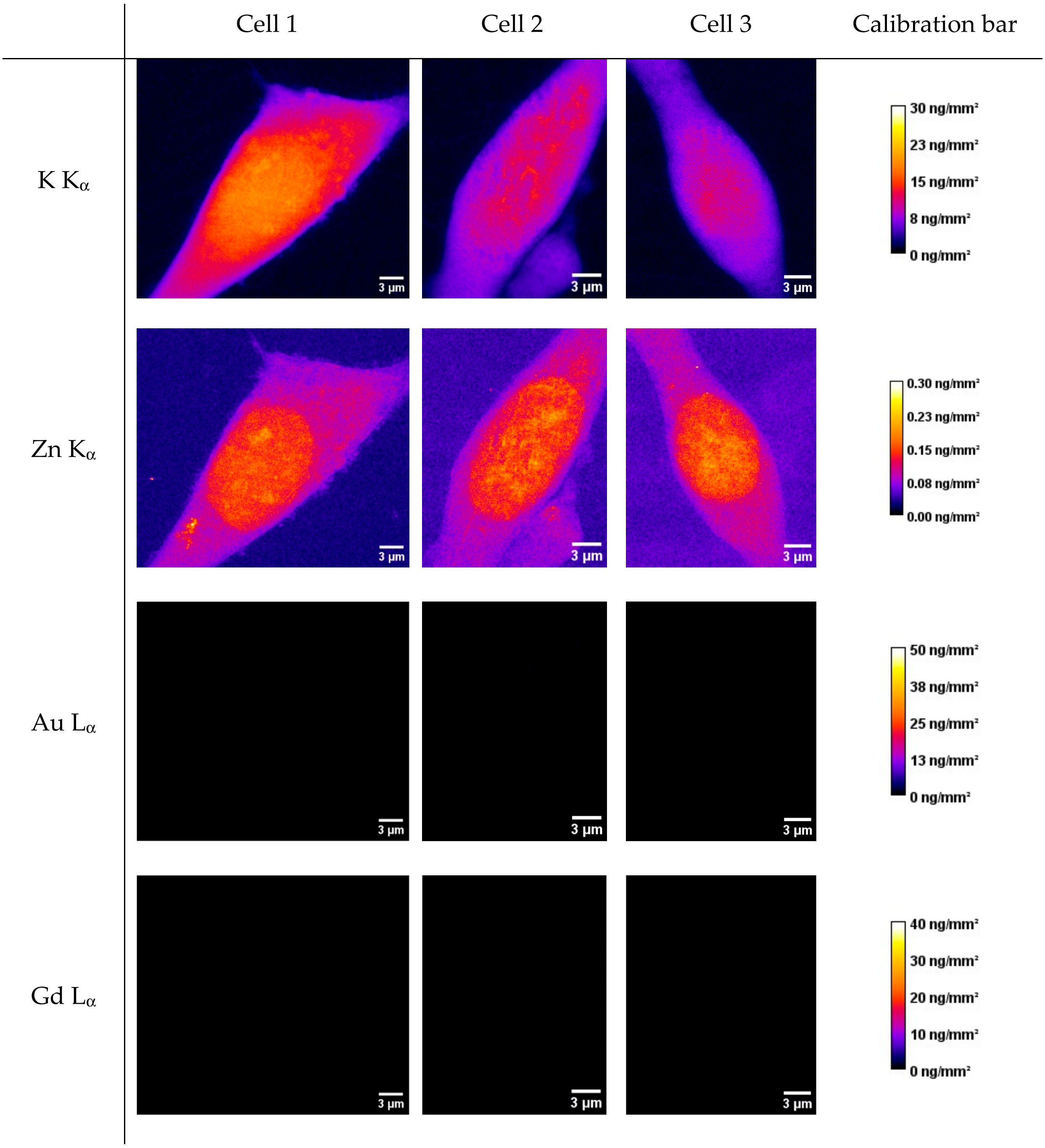

Figure S8 Pseudo colored images of 3T3 cells kept in AMEM growth media for 24 h acquired by XRF-imaging. The signals originated from: K K_α emission (first row), Zn K_α emission (second row), Au L_α emission (third row), and Gd L_α emission (fourth row). The scale bars indicate 3 µm.

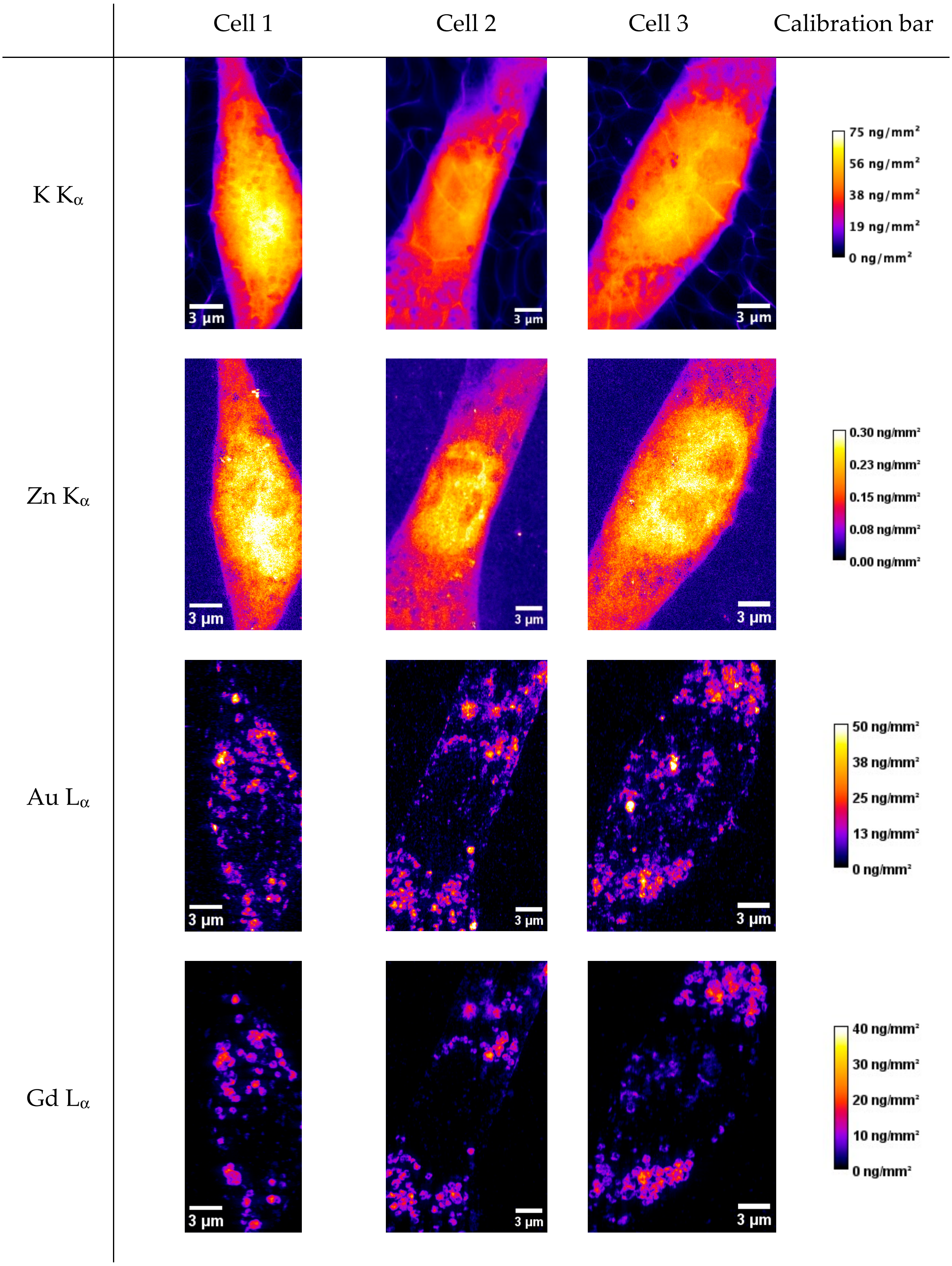

Figure S9 Pseudo colored images of 3T3 cells after 24 h of exposure with AuNP@CTPR-Gd (C_{Au} ~10 µg/mL), without incubation time in NP-free medium ($t_{inc} = 0$), acquired by XRF-imaging. The signals originated from: K K_α emission (first row), Zn K_α emission (second row), Au L_α emission (third row), and Gd L_α emission (fourth row). The scale bars indicate 3 µm.

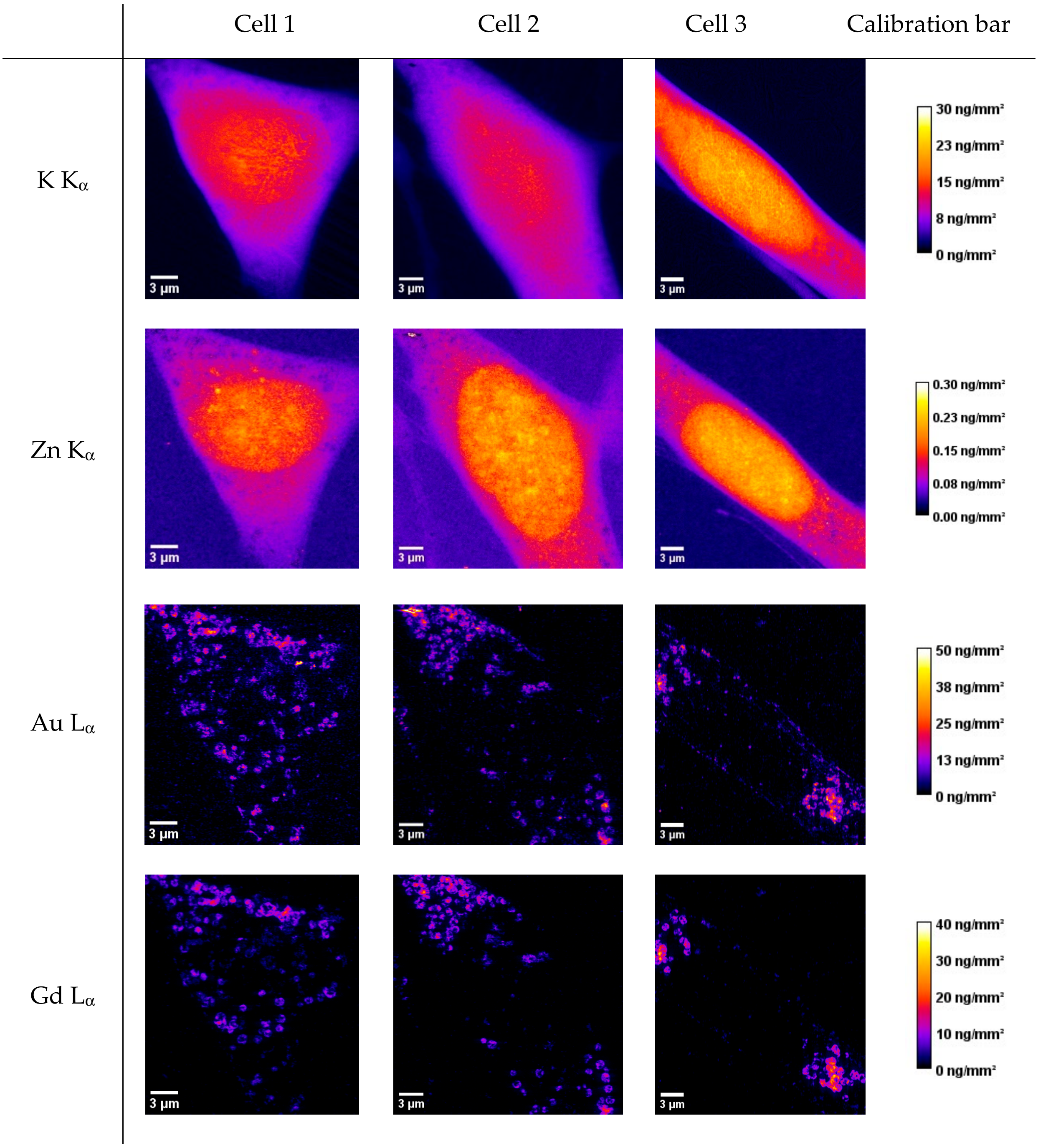

Figure S10 Pseudo colored images of 3T3 cells after 24 h of exposure with AuNP@CTPR-Gd (C_{Au} ~10 µg/mL) followed by t_{inc} = 30 min incubation time in NP-free medium, acquired by XRF-imaging. The signals originated from: K K_α emission (first row), Zn K_α emission (second row), Au L_α emission (third row), and Gd L_α emission (fourth row). The scale bars indicate 3 µm.

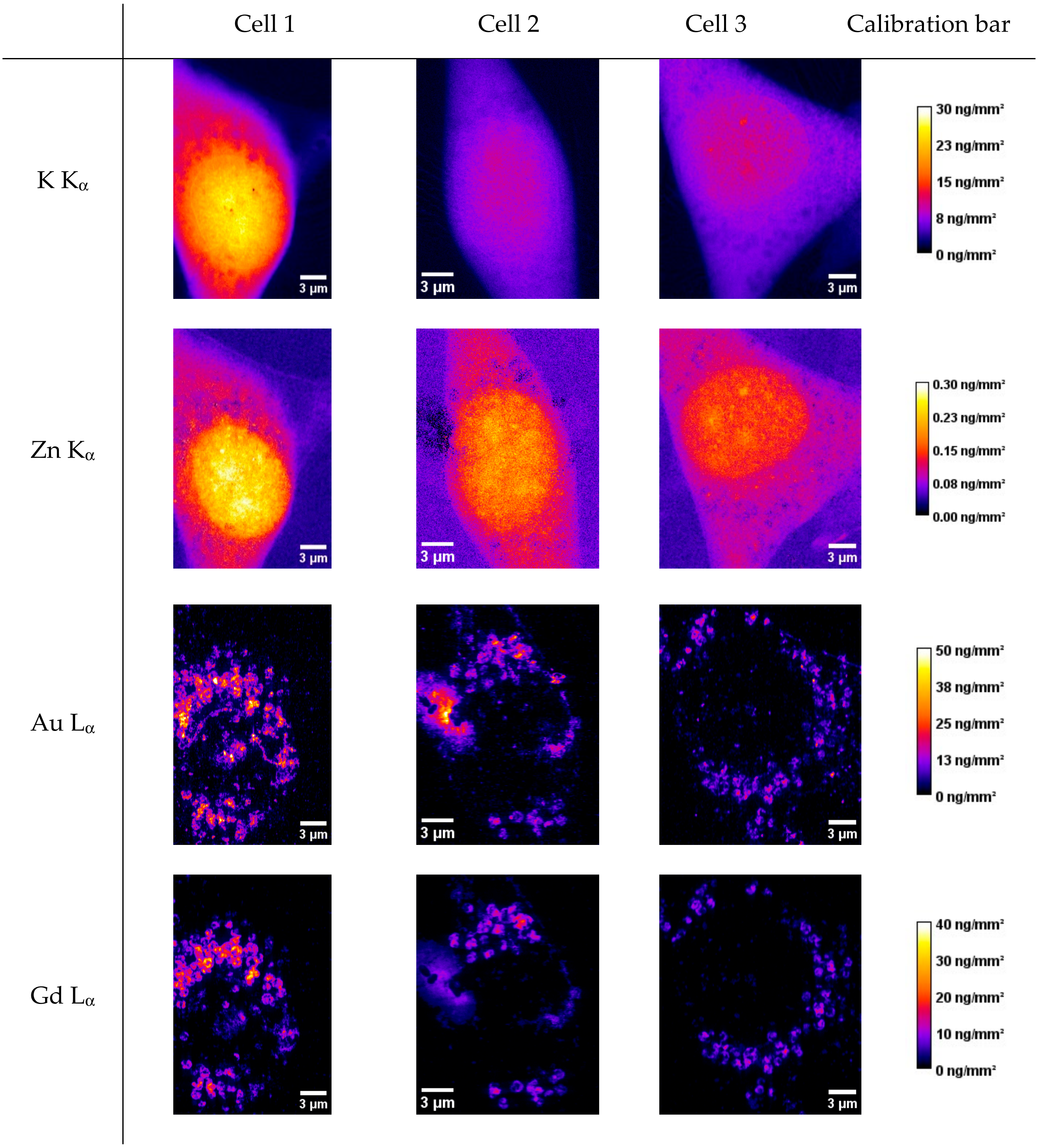

Figure S11 Pseudo colored images of 3T3 cells after 24 h of exposure with AuNP@CTPR-Gd (C_{Au} ~10 µg/mL) followed by t_{inc} = 60 min incubation time in NP-free medium, acquired by XRF-imaging. The signals originated from: K K_α emission (first row), Zn K_α emission (second row), Au L_α emission (third row), and Gd L_α emission (fourth row). The scale bars indicate 3 µm.

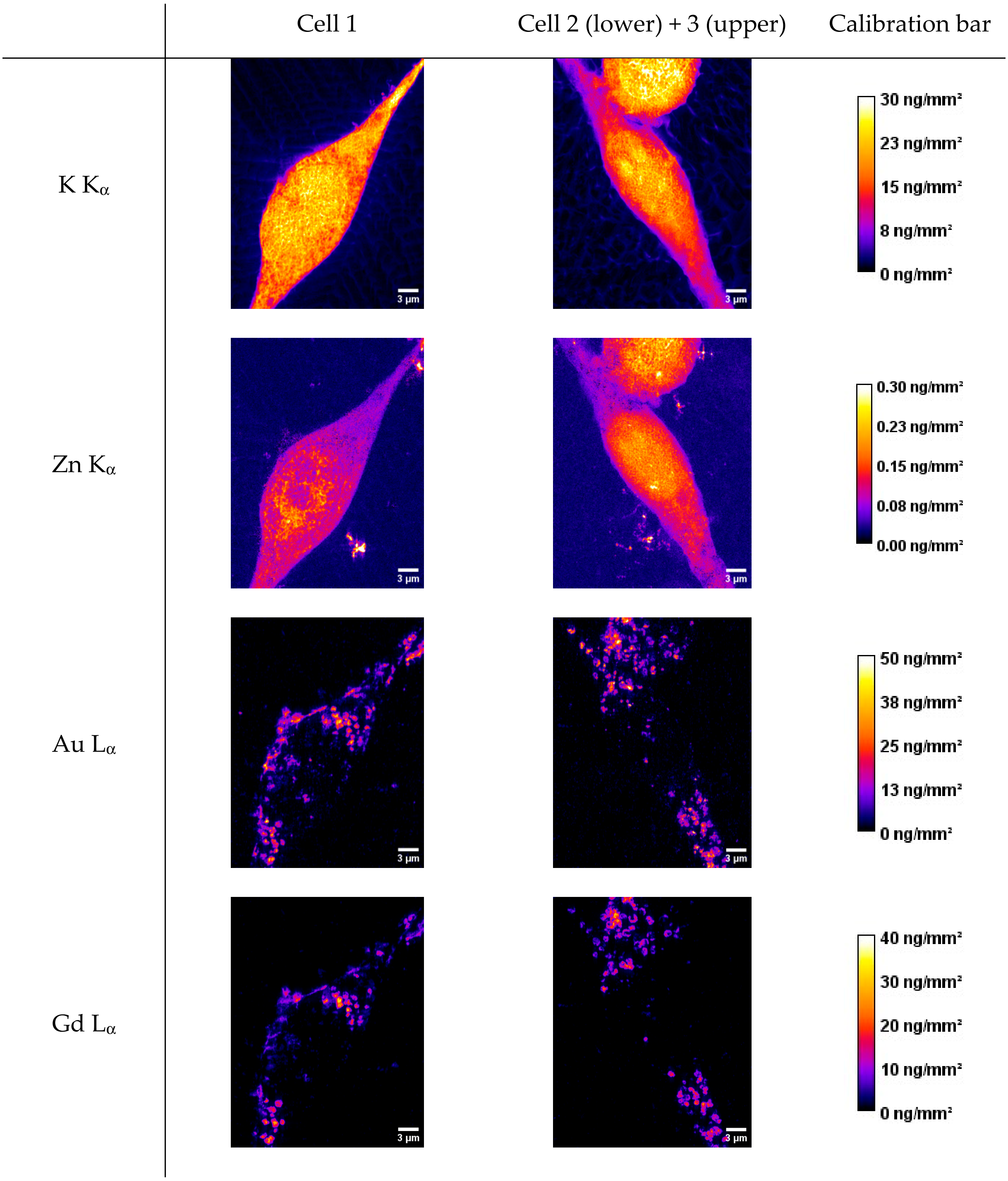

Figure S12 Pseudo colored images of 3T3 cells after 24 h of exposure with AuNP@CTPR-Gd (C_{Au} ~10 µg/mL) followed by t_{inc} = 120 min incubation time in NP-free medium, acquired by XRF-imaging. The signals originated from: K K_α emission (first row), Zn K_α emission (second row), Au L_α emission (third row), and Gd L_α emission (fourth row). The scale bars indicate 3 µm.

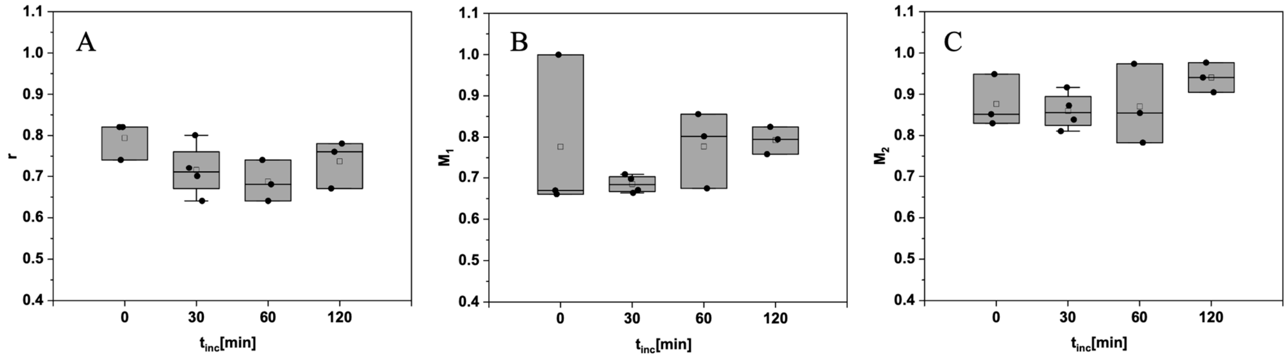

Figure S13 Colocalization between Au and Gd in 3T3 cells exposed to AuNP@CTPR-Gd (C_{Au} ~10 µg/mL) for 24 h, followed by different incubation times (t_{inc} = 0, 30, 60, and 120 min). Pearsons' correlation coefficient r (A), Manders' Coefficient M_1 (B) and M_2 (C) depicted in box plots.

S5.3. ROIs defined as vesicles

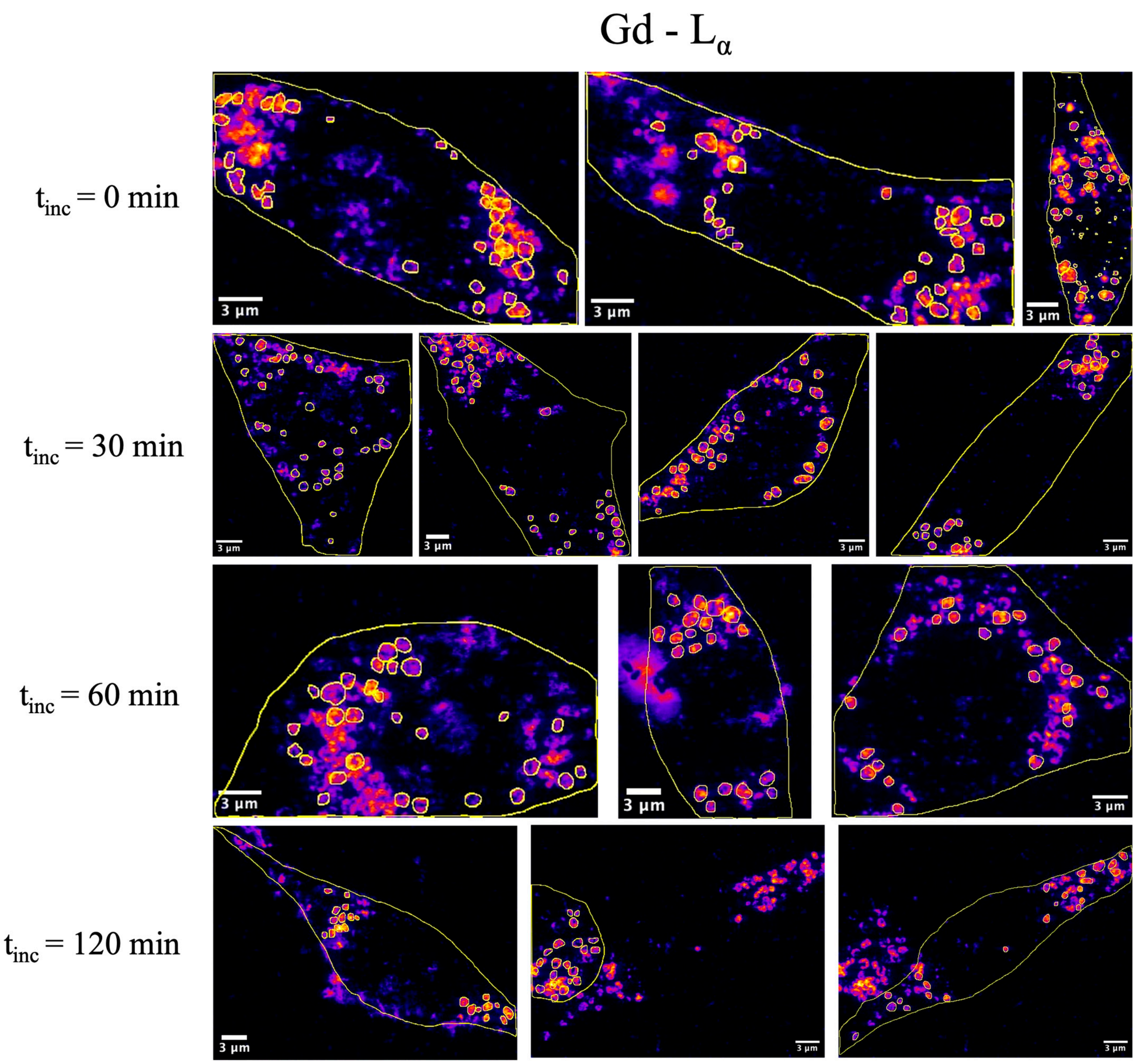

Figure S14 Pseudo colored images of 3T3 cells after 24 h of exposure to AuNP@CTPR-Gd (C_{Au} ~10 μg/mL), followed by t_{inc} = 0 min incubation time in NP-free medium (first row), t_{inc} = 30 min (second row), t_{inc} = 60 min (third row), and t_{inc} = 120 min (fourth row). The signal originated from Gd L_α emission. Regions of interest as outlined in yellow, show nearly spherical structures, smaller than 1 μm in diameter, which were used for analysis. The scale bars indicate 3 μm.

S5.4. ROIs in distance to cellular nucleus

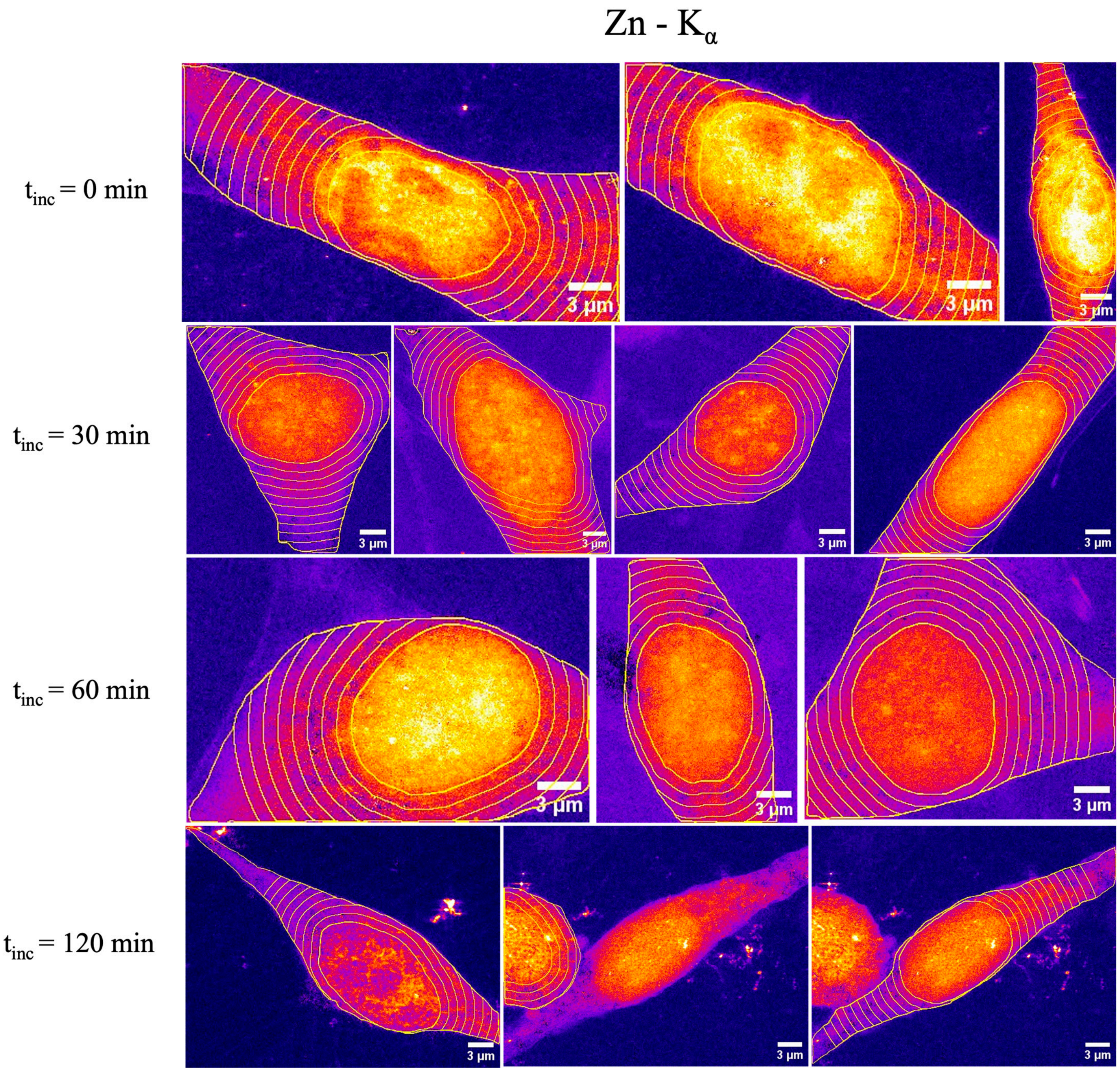

Figure S15 Pseudo colored images of 3T3 cells after 24 h of exposure to AuNP@CTPR-Gd (C_{Au} ~10 µg/mL), without additional incubation time t_{inc} = 0 (first row), with additional t_{inc} = 30 min incubation time in NP-free medium (second row), t_{inc} = 60 min (third row), or t_{inc} = 120 min (fourth row), respectively. The signal originated from Zn K_α emission. Yellow regions of interest show cellular nuclei (inner circle) and subsequent 1 µm distance steps inside cells. The scale bars indicate 3 µm.

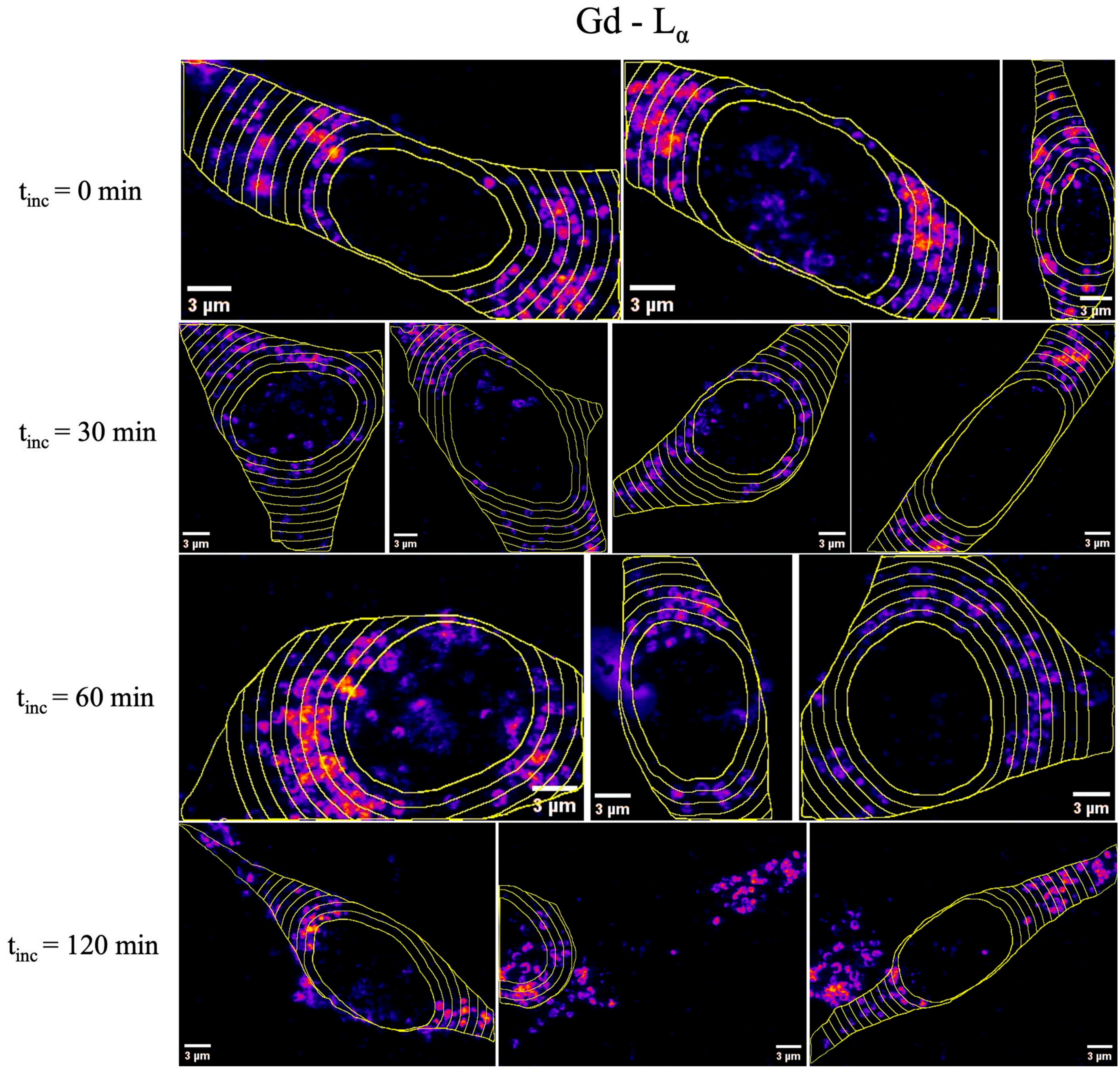

Figure S16 Pseudo colored images of 3T3 cells after 24 h of exposure to AuNP@CTPR-Gd (C_{Au} ~10 µg/mL), without additional incubation time t_{inc} = 0 (first row), with additional t_{inc} = 30 min incubation time in NP-free medium (second row), t_{inc} = 60 min (third row), or t_{inc} = 120 min (fourth row), respectively. The signal originated from Gd L_α emission. Yellow regions of interest show cellular nuclei (inner circle) and subsequent 1 µm distance steps inside cells. The scale bars indicate 3 µm.

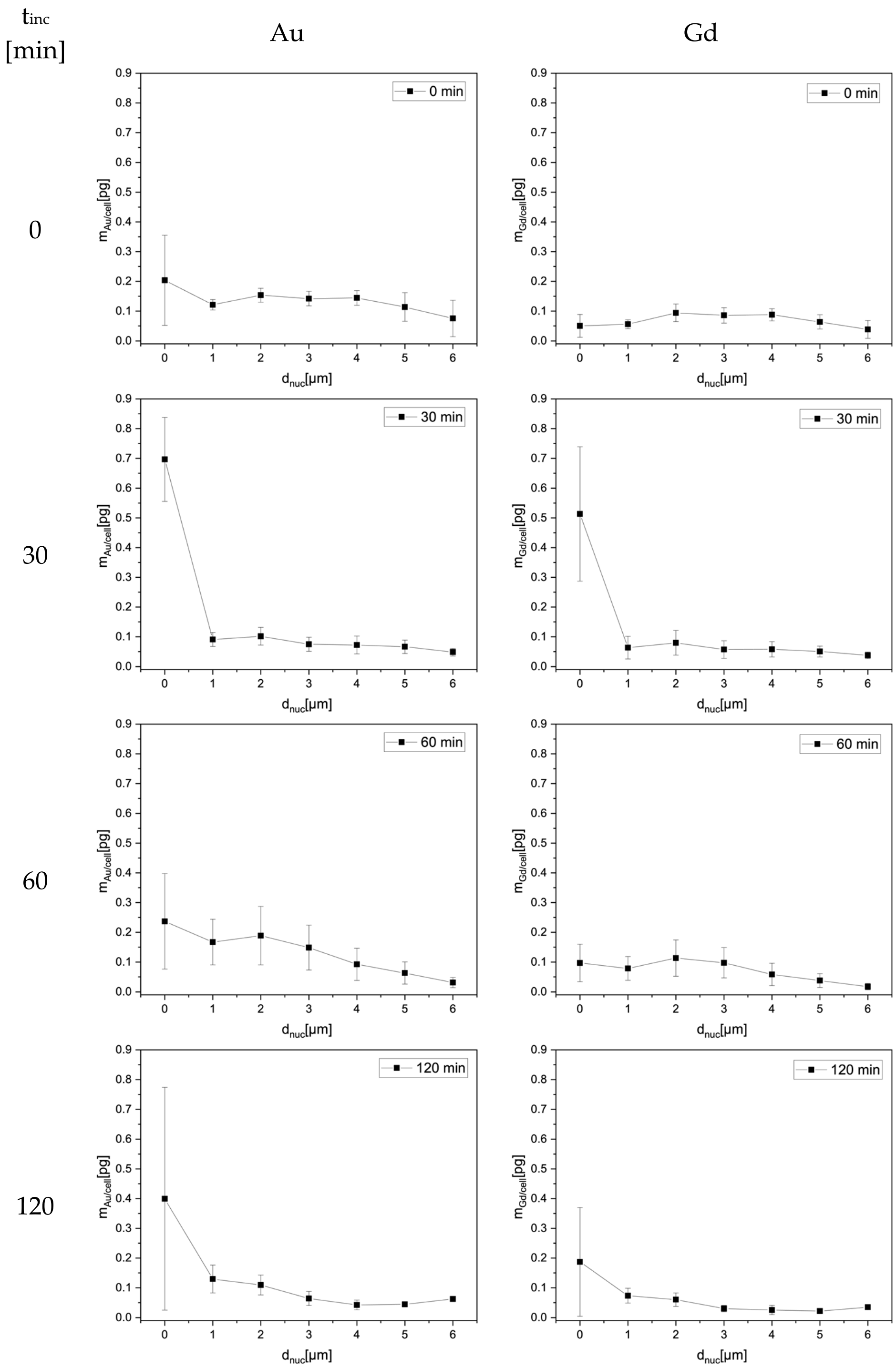

Figure S17 Quantities of Au ($m_{Au/cell}$, left) and Gd ($m_{Gd/cell}$, right) for different incubation times in NP-free medium t_{inc} as a function of the distance to the cellular nucleus d_{nuc}, as calculated from XRF-imaging maps.

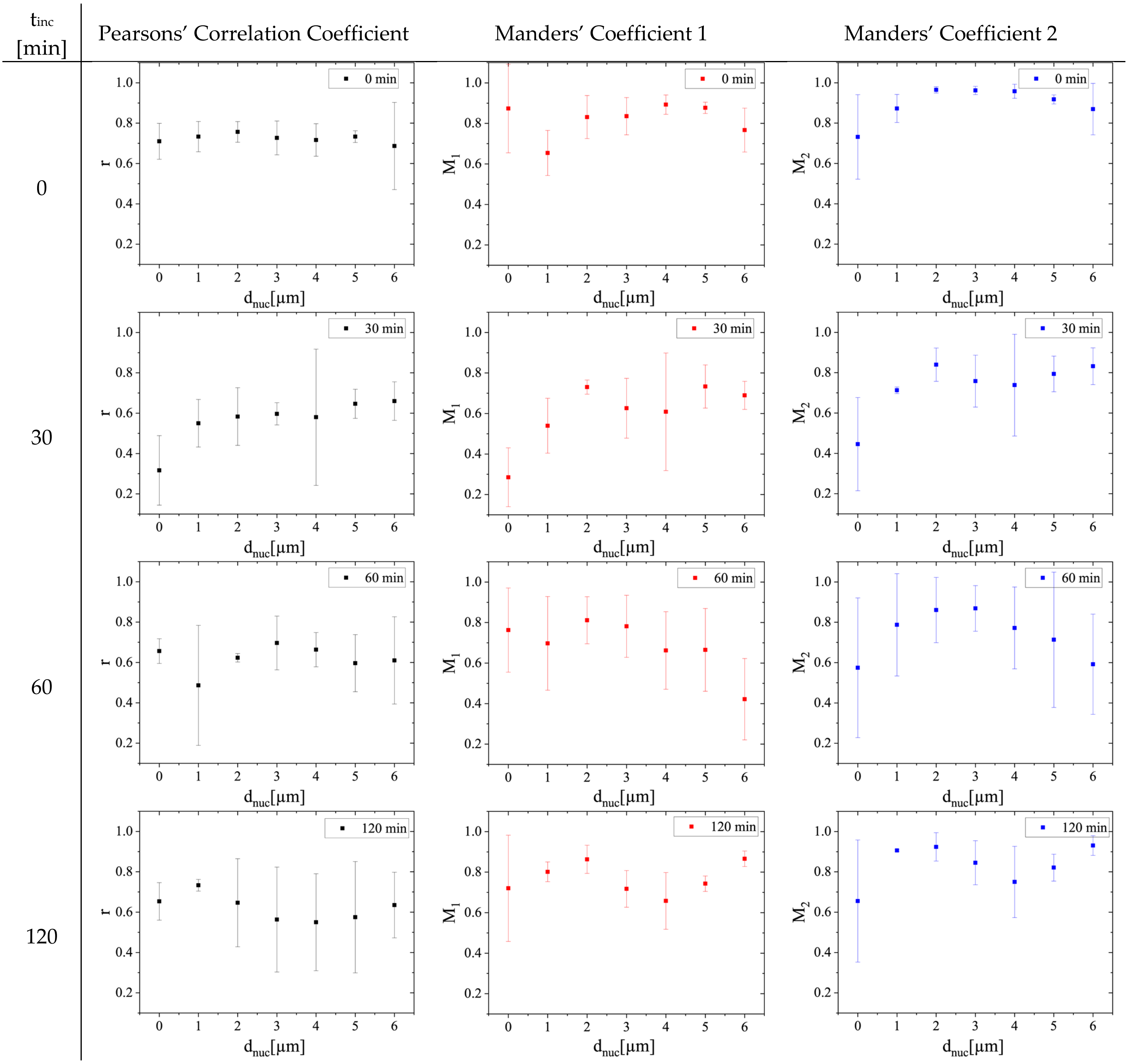

Figure S18 Colocalization between Au and Gd as a function of distance to the nucleus d_{nuc} in 3T3 cells exposed to AuNP@CTPR-Gd (C_{Au} ~10 µg/mL) followed by different incubation times in NP-free medium (t_{inc} = 0, 30, 60, and 120 min).

References

29 Schulz, F.; Homolka, T.; Bastús, N.G.; Puntes, V.; Weller, H.; Vossmeyer, T. Little Adjustments Significantly Improve the Turkevich Synthesis of Gold Nanoparticles. *Langmuir* **2014**, *30*, 10779–10784.

52 Schindelin, J.; Arganda-Carreras, I.; Frise, E.; Kaynig, V.; Longair, M.; Pietzsch, T.; Preibisch, S.; Rueden, C.; Saalfeld, S.; Schmid, B.; et al. Fiji: An Open-Source Platform for Biological-Image Analysis. *Nat Methods* 2012, *9*, 676–682.

53. Carril, M.; Padro, D.; del Pino, P.; Carrillo-Carrion, C.; Gallego, M.; Parak, W.J. In situ detection of the protein corona in complex Environments. *Nature Communications* **2017**, 8:1542, doi: 10.1038/s41467-017-01826-4.

54. Hühn, J.; Carrillo-Carrion, C.; Soliman, M.G.; Pfeiffer, C.; Valdeperez, D.; Masood, A.; Chakraborty, I.; Zhu, L.; Gallego, M.; Yue, Z.; et al. Selected Standard Protocols for the Synthesis, Phase Transfer, and Characterization of Inorganic Colloidal Nanoparticles. *Chemistry of Materials* **2017**, *29*, 399–461, doi:10.1021/acs.chemmater.6b04738.

55. Stockert, J.C.; Horobin, R.W.; Colombo, L.L.; Blázquez-Castro, A. Tetrazolium Salts and Formazan Products in Cell Biology: Viability Assessment, Fluorescence Imaging, and Labeling Perspectives. *Acta Histochem* 2018, *120*, 159–167.

56. Strober, W. Trypan Blue Exclusion Test of Cell Viability. *Curr Protoc Immunol* **2015**, *111*, A3.B.1-A3.B.3, doi:10.1002/0471142735.ima03bs111.

57. Liu, X.; Atwater, M.; Wang, J.; Huo, Q. Extinction Coefficient of Gold Nanoparticles with Different Sizes and Different Capping Ligands. *Colloids Surf B Biointerfaces* **2007**, *58*, 3–7, doi:10.1016/j.colsurfb.2006.08.005.

58. Milani, S.; Baldelli Bombelli, F.; Pitek, A.S.; Dawson, K.A.; Rädler, J. Reversible versus Irreversible Binding of Transferrin to Polystyrene Nanoparticles: Soft and Hard Corona. *ACS Nano* **2012**, *6*, 2532–2541, doi:10.1021/nn204951s.

59. Zhang, B.; Jin, H.; Li, Y.; Chen, B.; Liu, S.; Shi, D. Bioinspired Synthesis of Gadolinium-Based Hybrid Nanoparticles as MRI Blood Pool Contrast Agents with High Relaxivity. *J Mater Chem* **2012**, *22*, 14494–14501, doi:10.1039/c2jm30629h.

60. Pearson, K. Mathematical Contributions to the Theory of Evolution.—III. Regression, Heredity, and Panmixia. *Philosophical Transactions of the Royal Society of London. Series A, Containing Papers of a Mathematical or Physical Character* **1895**, *191*, 229–311.

61. Pike, J.A.; Styles, I.B.; Rappoport, J.Z.; Heath, J.K. Quantifying Receptor Trafficking and Colocalization with Confocal Microscopy. *Methods* **2017**, *115*, 42–54, doi:10.1016/j.ymeth.2017.01.005.

62. Bolte, S.; Cordelières, F.P. A Guided Tour into Subcellular Colocalization Analysis in Light Microscopy. *J Microsc* **2006**, *224*, 213–232, doi:10.1111/j.1365-2818.2006.01706.x.

63. Manders, E.M.M.; Verbeek, F.J.; Aten, J.A. Measurement of Co-localization of Objects in Dual-colour Confocal Images. *J Microsc* **1993**, *169*, 375–382, doi:10.1111/j.1365-2818.1993.tb03313.x.

64. Aaron, J.S.; Taylor, A.B.; Chew, T.L. Image Co-Localization - Co-Occurrence versus Correlation. *J Cell Sci* **2018**, *131*, doi:10.1242/jcs.211847.

65. Costes, S. V.; Daelemans, D.; Cho, E.H.; Dobbin, Z.; Pavlakis, G.; Lockett, S. Automatic and Quantitative Measurement of Protein-Protein Colocalization in Live Cells. *Biophys J* **2004**, *86*, 3993–4003, doi:10.1529/biophysj.103.038422.
